# New possibilities for egg white lysozyme: heat-denatured lysozyme partially inactivates select foot-and-mouth disease virus strains

**DOI:** 10.1038/s41598-020-80239-8

**Published:** 2021-01-12

**Authors:** Katsuhiko Fukai, Kazuyuki Inoue, Akira Takeuchi, Makoto Yamakawa

**Affiliations:** 1 Exotic Disease Research Station, National Institute of Animal Health, National Agriculture and Food Research Organization, 6-20-1 Josui-honcho, Kodaira, Tokyo, 187-0022 Japan; 2 Functional Materials Department, Institute of Technology Solutions, R&D Division, Kewpie Corporation, Sengawa Kewport, 2-5-7 Sengawa-cho, Chofu, Tokyo, 182-0002 Japan

**Keywords:** Virology, Antivirals

## Abstract

Foot-and-mouth disease (FMD) is one of the most contagious diseases of cloven-hoofed animals. Disinfectants are used to inactivate FMD virus (FMDV) in Japan. Reports that heat-denatured lysozyme inactivates bacteria as well as viruses, such as norovirus and hepatitis A virus, led us to determine its effects on FMDV. We show here that heat-denatured lysozyme partially inhibited the infectivity of FMDV O/JPN/2010-1/14C but of FMDVs A/TAI/46-1/2015 and Asia1/Shamir (ISR/3/89). Further, heat-denatured lysozyme variably reduced RNA loads of FMDVs O/JPN/2010-1/14C, O/MOG/2/Ca/BU/2017, O/Taiwan/1997, Asia1/Shamir (ISR/3/89), Asia1/TUR/49/2011, SAT1/KEN/117/2009, SAT2/SAU/6/2000 and SAT3/ZIM/3/83 but could not those of O/JPN/2000, A/TAI/46-1/2015, A_22_/IRQ/24/64, A_15_/TAI/1/60 and C/PHI/7/84. These findings indicate that heat-denatured lysozyme may serve as a new disinfectant against FMDV.

## Introduction

Foot-and-mouth disease (FMD) is the most contagious disease of cloven-hoofed domestic and wild animals, including cattle, water buffalo, sheep, goats and pigs^1^. FMD virus (FMDV), a member of the genus *Aphthovirus* within the family *Picornaviridae*, causes FMD^1^. The serotypes of FMDV are as follows: A, O, C, Asia1, SAT1, SAT2 and SAT3^1^. Topotypes (cut-off, 80–85% VP1 nucleotide [nt] sequence identities), lineages (approximately 90% VP1 nt identity) and sublineages (approximately 95% VP1 nt identity) define the phylogenetic clustering of VP1 sequences of each serotype^2^.

Lysozyme hydrolyses peptidoglycans, which are components of the cell walls of gram-positive bacteria^3^. Lysozyme is present in human tears and breast milk and in avian egg white^3^. Moreover, hen’s eggs are the richest source of lysozyme, accounting for 3.5% of egg white protein^4^. Egg white lysozyme is a natural antibacterial agent categorized as Generally Recognized as Safe by the United States Food and Drug Administration and therefore is used in the food industry as well as in pharmaceutical science^3^. Commercial egg white lysozyme controls bacterial contamination of meat products such as sausages, salami, pork, beef, and turkey as well as to prevent the growth of *Clostridium tyrobutyricum* during cheese-making, and to control the growth of lactic bacteria in wine and beer production. Further, egg white lysozyme is used in cosmetic applications and pharmaceuticals^4^.

Heat-denatured lysozyme possesses a broader antibacterial spectrum than native lysozyme^5^ and inhibits gram-positive and gram-negative bacteria^5^. The broader antibacterial effects of heat-denatured lysozyme may be conferred by conformational changes and the amino acid sequences of specific peptides^5^. Moreover, native and heat-denatured lysozymes inactive herpes simplex virus and human immunodeficiency virus^6,7^ and heat-denatured lysozyme inhibits murine norovirus (MNV) and hepatitis A virus^8–10^.

The Ministry of Agriculture, Forestry and Fisheries of Japan recommends the disinfectants 4% sodium carbonate, hydrated lime and other commercial products to inactivate FMDV^11^. However, as mentioned above, heat-denatured lysozyme may serve the same purpose. Here we aimed to evaluate the disinfectant activity of heat-denatured lysozyme against FMDV and the implications of our findings for preventing FMD.

## Results

### Inactivation of FMDV infectivity by heat-denatured lysozyme

**Figure 1:**
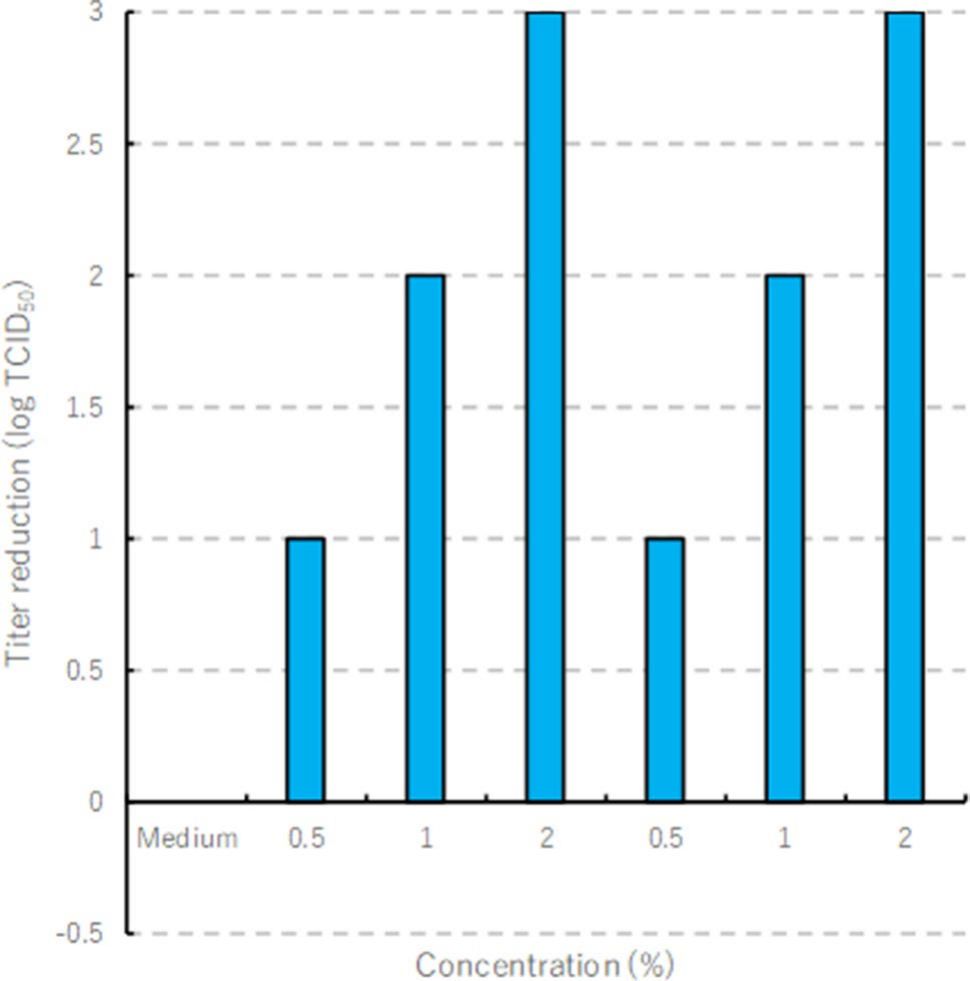
Relationship between the concentration of the heat-denatured lysozyme and inactivation of FMDV O/JPN/2010-1/14C.

Solutions of 0.25%, 0.5%, 1% and 2% (pH 6.5 and 7.0) of heat-denatured lysozyme reduced titers of FMDV O/JPN/2010-1/14C by 10^1^, 10^1^, 10^2^ and 10^3^ TCID_50_ units, respectively (Fig. 1). In contrast, these same conditions did not significantly reduce titers of FMDVs A/TAI/46-2/2015 and Asia1/Shamir (ISR/3/89) (data not shown).

### Reduction of FMDV RNA loads by the heat-denatured lysozyme

**Figure 2:**
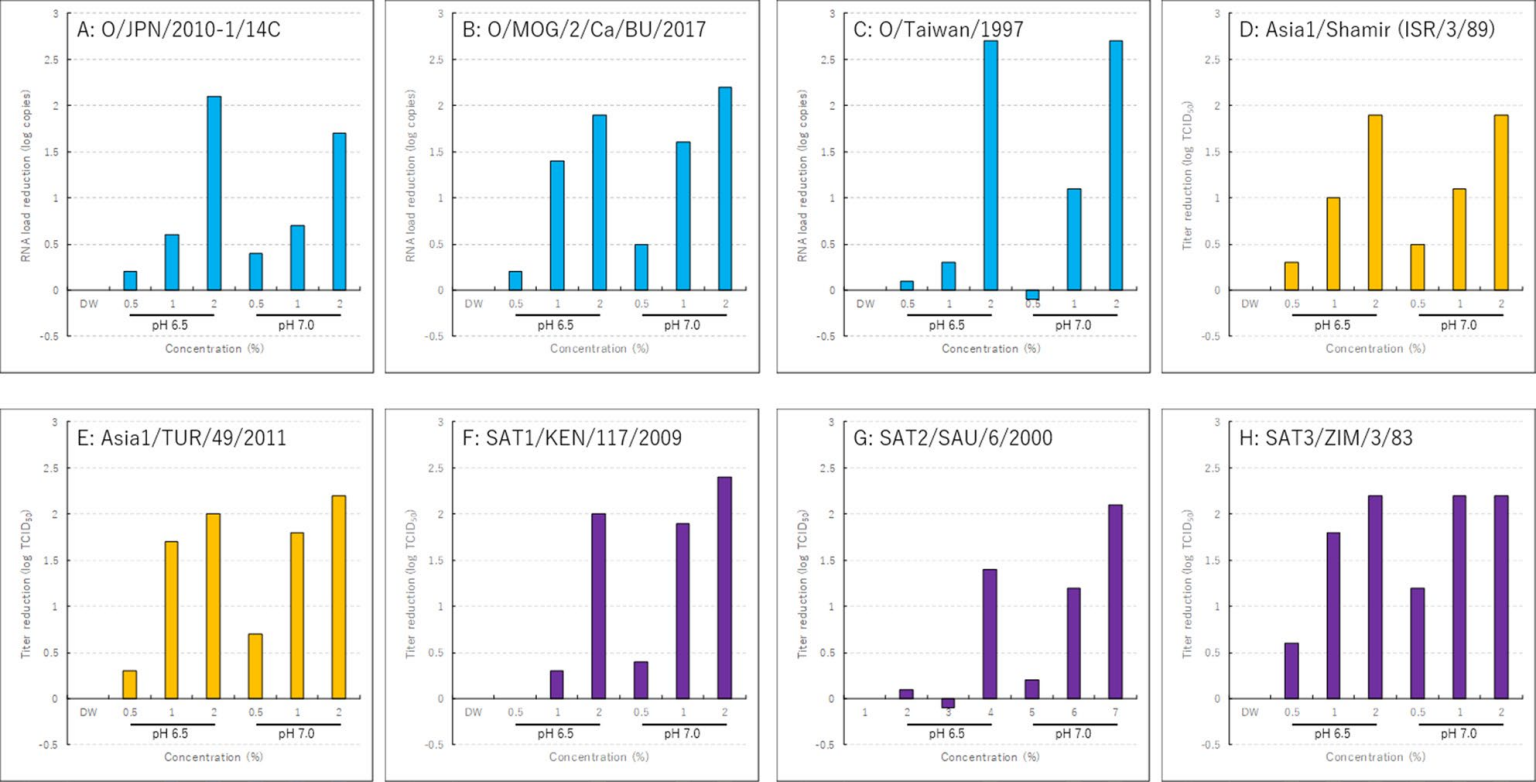
Relationship between concentration of the heat-denatured lysozyme and the RNA load of FMDVs. (**A**) O/JPN/2010-1/14C, (**B**) O/MOG/2/Ca/BU/2017, (**C**) O/Taiwan/1997, (**D**) Asia1/Shamir (ISR/3/89), (**E**) Asia1/TUR/49/2011, (**F**) SAT1/KEN/117/2009, (**G**) SAT2/SAU/6/2000, (**H**) SAT3/3/83.

Solutions of 0.5%, 1% and 2% (pH 6.5 and 7.0) of heat-denatured lysozyme variably reduced RNA loads of FMDVs O/JPN/2010-1/14C, O/MOG/2/Ca/BU/2017, O/Taiwan/1997, Asia1/Shamir (ISR/3/89), Asia1/TUR/49/2011, SAT1/KEN/117/2009, SAT2/SAU/6/2000 and SAT3/ZIM/3/83 (Fig. 2). In contrast, the RNA loads of FMDVs O/JPN/2000, A/TAI/46-1/2015, A_22_/IRQ/24/64, A_15_/TAI/1/60 and C/PHI/7/84 were not significantly reduced (data not shown).

## Discussion

Lysozyme degrades the bacterial cell walls^3^ and is mainly against gram-positive bacteria^3,12^. Heat-denaturation of lysozyme changes its conformation, and its antimicrobial spectrum includes gram-negative bacteria and others^5,13^. Although heat-denaturation inactivates the enzymatic activity of lysozyme, specific constituent amino acids may be affected, which confer antimicrobial properties^5^. Further, heat-denatured lysozyme inactivates MNV and hepatitis A virus^8–10^. Here we show that heat-denatured lysozyme inactivated FMDV. Specifically, heat-denatured lysozyme inhibited the infectivity of FMDV O/JPN/2010-1/14C, in a concentration-dependent manner, by as much as 10^3^ TCID_50_ units.

In Japan, 4% sodium carbonate solution and several commercial products are generally used as disinfectants to inactivate FMDV^11^. However, the effects of these disinfectants are reduced by contact with organic matter^14^, which limits their use in food production. When these disinfectants are used to sterilize food products, they must be washed or treated before consumption for safety purposes. In contrast, heat-denatured lysozyme is safer than these disinfectants because lysozyme is derived from egg white. To our knowledge, this is the first report to show that heat-denatured lysozyme can partially reduce the infectivity of FMDV.

Propidium monoazide (PMA) enters damaged cells and inhibits PCR assays by binding to nucleic acids^15^. Further, through the same mechanism, PMA is used to effectively measure virus enumeration^16^. Here we used real-time RT-PCR combined with PMA to measure the reduction of viral RNA loads of FMDV strains treated with heat-denatured lysozyme. We found that heat-denatured lysozyme reduced the viral RNA loads of FMDV O/Taiwan/1997 by as much as 2.7 logs. The viral RNA loads of several FMDVs were variably reduced as well. However, the results acquired using real-time RT-PCR combined with PMA did not completely correlate with findings of reduced viral infectivity. These results may be explained by findings that PMA cannot completely bind all nucleic acids^17^.

The mechanism of inactivation of FMDV by heat-denatured lysozyme is unknown. However, assays employing real-time RT-PCR combined with PMA show that heat-denatured lysozyme disrupts the capsid proteins of FMDV, which may contribute to the mechanism of the inhibitory effects of heat-denatured lysozyme. For example, the size of MNV incubated with heat-denatured lysozyme expanded compared with that of unincubated MNV or MNV incubated with native lysozyme^8^. Therefore, changes of the conformations of capsid proteins increase the size of the virion, which accounts for the mechanism of inactivation of MNV by heat-denatured lysozyme. In addition, antiviral activity of lysozyme appears to be independent from its bacteriolytic activity, and is related to its positive charge that induces instability in cellular membranes^6,18^. Egg white lysozyme stops the typical cell fusion induced by herpes simplex virus; however, addition of negative charges to this molecule drastically reduced its antiviral activity^18^. Furthermore, the hydrophobicity of heat-denatured lysozyme tends to increase as the pH increased and contributes to MNV inactivation by heat-denatured lysozyme^19^. Further investigations are required to determine whether FMDV is inactivated through a similar mechanism.

The amino acid sequences of lysozyme and α-lactalbumin are approximately 70% identical^20^, which is reflected by their similar secondary structures. Although the structural properties of lysozyme and α-lactalbumin are similar (secondary and tertiary structures, constituent amino acid ratios, and specific amino acid regions), the latter does not inactivate MNV^8^. These results suggest that inactivation of MNV by heat-denatured lysozyme is caused by a specific domain within lysozyme, rather than its entire structure. When the amino acid sequence of lysozyme was divided into three regions according to its secondary structure, one polypeptide inhibited MNV significantly more than the other peptides. Further, there is no significant difference between the inhibitory activities of the native and heat-denatured peptides, indicating that the primary amino acid sequence is responsible for inactivation of MNV. In addition, heat denaturation may also change the conformation of lysozyme, which confers its inhibitory activity. In contrast, α-lactalbumin lacks a corresponding sequence, which likely accounts for its inability to inactivate MNV.

Here we show that heat-denatured lysozyme did not significantly reduce the infectivities of FMDVs A/TAI/46-1/2015 and Asia1/Shamir (ISR/3/89), and it did not sufficiently reduce the RNA loads of FMDVs O/JPN/2000, A/TAI/46-1/2015, A_22_/IRQ/24/64, A_15_/TAI/1/60 and C/PHI/7/84. These FMDV strains are not identical; however, heat-denatured lysozyme reduced the titers/RNA loads of serotype O strains O/JPN/2010-1/14C, O/MOG/2/Ca/BU/2017 and O/Taiwan/1997, but not those of O/JPN/2000, and it did reduced the RNA loads of Asia1, SAT1, SAT2 and SAT3 FMDVs to varying extents. Therefore, heat-denatured lysozyme cannot be used alone as a disinfectant for FMDV; however, it may be used to supplement other substances to inactivate FMDV. Therefore, comparative analyses of nucleotide and amino acid sequences of FMDV strains may disclose key determinants of heat-denatured lysozyme that inactivate FMDV. Further investigations are therefore required to improve the ability of heat-denatured lysozyme to inactivate FMDV.

## Methods

### Cells and viruses

IB-RS-2^21^, BHK-21^22^ and LFBK-αvβ6 cells^23^ were maintained at 37 °C using Dulbecco’s Modified Eagle Medium/Nutrient Mixture F12 (Thermo Fisher Scientific, Waltham, MA, USA) supplemented with 10% fetal bovine serum (FUJIFILM Wako Chemicals Corporation, Osaka, Japan).

FMDV O/JPN/2010-1/14C (SEA topotype, Mya-98 lineage, GenBank: LC149617)^24^, O/JPN/2000 (ME-SA topotype, PanAsia lineage, GenBank: AB079061 and AB079062)^25^, O/MOG/2/Ca/BU/2017 (ME-SA topotype, Ind-2001e lineage, GenBank: LC320038)^26^, O/Taiwan/97 (CATHAY topotype, GenBank: AF308157)^27^, A/TAI/46-1/2015 (ASIA topotype, Sea-97 lineage, GenBank: LC564917), A_22_/IRQ/24/64 (ASIA topotype, A_22_ lineage, GenBank: AY593763), A_15_/TAI/1/60 (ASIA topotype, A_15_ lineage, GenBank: AY593755), C/PHI/7/84 (EURO-SA topotype, GenBank: KY091303), Asia1/Shamir (ISR/3/89) (ASIA topotype, GenBank: JF739177), Asia1/TUR/49/2011 (ASIA topotype, Sindh-08 lineage)^28^, SAT1/KEN/117/2009 (I (NWZ) topotype), SAT2/SAU/6/2000 (VII topotype, GenBank: AF367135) and SAT3/ZIM/3/83 (I (SEZ) topotype) were produced using monolayers of IB-RS-2 and BHK-21 cells. FMDV O/JPN/2010-1/14C and O/JPN/2000 strains were isolated from 2010 and 2000 epidemics in Japan, respectively^24,25^. FMDV O/MOG/2/Ca/BU/2017, O/Taiwan/97 and A/TAI/46-1/2015 strains were kindly supplied from State Central Veterinary Laboratory (Mongolia), Animal Health Research Institute (Taiwan) and Regional Reference Laboratory for Foot and Mouth Disease in South East Asia (Thailand), respectively. FMDV A_22_/IRQ/24/64, A_15_/TAI/1/60, C/PHI/7/84, Asia1/Shamir (ISR/3/89), Asia1/TUR/49/2011, SAT1/KEN/117/2009, SAT2/SAU/6/2000 and SAT3/ZIM/3/83 strains were obtained from The Pirbright Institute (UK).

### Inactivation of the infectivity of FMDV by the heat-denatured lysozyme

Egg white lysozyme was dissolved at concentration of 4% (w/v) in distilled water (pH 6.5 and 7.0) and denatured in a water bath for 30 min at 100 °C. The solution of 4% of heat-denatured lysozyme was further diluted serially twofold from 2 to 0.5% by distilled water. FMDVs O/JPN/2010-1/14C, A/TAI/46-1/2015 and Asia1/Shamir (ISR/3/89) were treated as follows: equal volumes of FMDV and heat-denatured lysozyme were mixed, placed at room temperature for 1 min and then immediately diluted tenfold with Dulbecco’s Modified Eagle Medium/Nutrient Mixture F12 supplemented with 10% fetal bovine serum to stop inactivation by the heat-denatured lysozyme. Virus titers were determined using LFBK-αvβ6 cells as described previously^29,30^.

### Reduction of viral RNA loads of FMDV by heat-denatured lysozyme

The FMDV strains listed above were treated as follows: equal volumes of FMDV and the heat-denatured lysozyme were mixed and placed at room temperature for 1 min and then immediately diluted tenfold with Dulbecco’s Modified Eagle Medium/Nutrient Mixture F12 supplemented with 10% fetal bovine serum. PMA (Biotium, Hayward, CA, USA) was added to the mixture to 50 μM. The mixture was incubated in the dark for 5 min at room temperature and then irradiated for 15 min using a 375 nm UV-light source. Viral RNAs were extracted from the mixture using a High Pure Viral RNA Kit (Roche Diagnostics, Basel, Switzerland) according to the manufacturer’s instructions. Real-time RT-PCR assays were conducted using TaqMan Fast Virus 1-Step Master Mix (Thermo Fisher Scientific) with primer-set 3D Forward (5′-ACTGGGTTTTACAAACCTGTGA-3′) and 3D Reverse (5′-GCGAGTCCTGCCACGGA-3′), and 3D Probe (5′-TCCTTTGCACGCCGTGGGAC-3′) as previously described^29,31^. Viral RNA loads were determined by comparison with a standard curve prepared from a positive amplification control containing a segment of the 3D gene of O/JPN/2010-1/14C. The control sequence was transcribed using the T7 promoter with an mMESSAGE mMACHINE T7 Ultra Kit (Thermo Fisher Scientific) according to the manufacturer’s instructions. The resulting preparation was purified using extraction with a mixture of phenol:chloroform:isoamyl alcohol (25:24:1) and then chloroform and precipitated using isopropanol. The RNA preparations were subjected to RT-PCR in the absence of reverse transcriptase to confirm the complete removal of contaminating DNAs. The RNAs were then quantified using an Ultrospec 2100 Pro (GE Healthcare Bio-Sciences, Pittsburgh, PA, USA). Serial ten-fold dilutions of the RNA preparation were used to generate a standard curve for real-time RT-PCR.
